# A Pilot Study of an mHealth Application for Healthcare Workers: Poor Uptake Despite High Reported Acceptability at a Rural South African Community-Based MDR-TB Treatment Program

**DOI:** 10.1371/journal.pone.0064662

**Published:** 2013-05-28

**Authors:** Krisda H. Chaiyachati, Marian Loveday, Stephen Lorenz, Neal Lesh, Lee-Megan Larkan, Sandro Cinti, Gerald H. Friedland, Jessica E. Haberer

**Affiliations:** 1 Yale University School of Medicine, New Haven, Connecticut, United States of America; 2 Tugela Ferry Care and Research Collaboration (TF CARES), Tugela Ferry, South Africa; 3 Health Systems Research Unit, Medical Research Council, Cape Town, South Africa; 4 Discipline of Public Health Medicine, University of KwaZulu-Natal, Durban, South Africa; 5 Laboratory of Computer Science, Massachusetts General Hospital, Boston, Massachusetts, United States of America; 6 Dimagi, Inc., Charlestown, Massachusetts, United States of America; 7 KwaZulu-Natal Department of Health, Greytown, South Africa; 8 University of Michigan Medical School & Veterans Affairs Ann Arbor Health System, Ann Arbor, Michigan, United States of America; 9 Massachusetts General Hospital, Harvard Medical School, Boston, Massachusetts, United States of America; McGill University, Canada

## Abstract

**Introduction:**

As the South African province of KwaZulu-Natal addresses a growing multidrug-resistant tuberculosis (MDR-TB) epidemic by shifting care and treatment from trained specialty centers to community hospitals, delivering and monitoring MDR-TB therapy has presented new challenges. In particular, tracking and reporting adverse clinical events have been difficult for mobile healthcare workers (HCWs), trained health professionals who travel daily to patient homes to administer and monitor therapy. We designed and piloted a mobile phone application (*Mobilize*) for mobile HCWs that electronically standardized the recording and tracking of MDR-TB patients on low-cost, functional phones.

**Objective:**

We assess the acceptability and feasibility of using *Mobilize* to record and submit adverse events forms weekly during the intensive phase of MDR-TB therapy and evaluate mobile HCW perceptions throughout the pilot period.

**Methods:**

All five mobile HCWs at one site were trained and provided with phones. Utilizing a mixed-methods evaluation, mobile HCWs’ usage patterns were tracked electronically for seven months and analyzed. Qualitative focus groups and questionnaires were designed to understand the impact of mobile phone technology on the work environment.

**Results:**

Mobile HCWs submitted nine of 33 (27%) expected adverse events forms, conflicting with qualitative results in which mobile HCWs stated that *Mobilize* improved adverse events communication, helped their daily workflow, and could be successfully expanded to other health interventions. When presented with the conflict between their expressed views and actual practice, mobile HCWs cited forgetfulness and believed patients should take more responsibility for their own care.

**Discussion:**

This pilot experience demonstrated poor uptake by HCWs despite positive responses to using mHealth. Though our results should be interpreted cautiously because of the small number of mobile HCWs and MDR-TB patients in this study, we recommend carefully exploring the motivations of HCWs and technologic enhancements prior to scaling new mHealth initiatives in resource poor settings.

## Introduction

South Africa’s health system is challenged by a multidrug-resistant tuberculosis (MDR-TB) incidence rate of 70 per 100,000 people amidst a TB-HIV co-infection proportion of 65%, one of the highest in the world [1]–[3]. High-burdened provinces like KwaZulu-Natal (KZN) began shifting care in 2007 from a hospital-based MDR-TB referral system to community-based treatment centers, because referral hospitals could not accommodate the overwhelming demand for inpatient therapy initiation. Moreover, patients traveled days for follow-up appointments and medication refills, making medication adherence and retention in care difficult [4]–[6].

Decentralizing care has expanded MDR-TB treatment access to rural parts of KZN; however, this model of MDR-TB care continues to struggle within a resource-strained health system [7]. KZN’s TB control program often faces drug shortages and insufficient funds for personnel like mobile healthcare workers (HCWs) who spend hours traversing poorly paved roads and rocky terrain to administer injectable agents daily and conduct in-home clinical assessments [4], [5], [8]. Effective clinical management and monitoring have both suffered amidst resource constraints and as a result mobile HCWs receive inadequate training and field support for clinical decision-making.

In particular, tracking and addressing adverse events – medication side effects or clinical deterioration – has been inadequate [7], [9]. Mobile HCWs have been asked to describe symptoms on traditional paper forms in the field and then submit them–a process that rarely happens. Moreover, symptom descriptions can be vague, captured as either occurring or not, and lack the level of detail sufficient for physicians to make clinical decisions. Even after physician recommendations are made, plans often take weeks to implement because of the inefficient communication process. Monitoring for adverse events is not only critical for patient safety but may also improve medication adherence [10]–[15].

From 2010 to 2011, we designed and systematically piloted an mHealth (mobile technologies for health) intervention, called *Mobilize*, as one potential solution for improving the acceptability feasibility of clinical monitoring and management of adverse events in patients receiving community-based MDR-TB treatment. The application of mHealth in low and middle-income countries has been recognized as a promising, creative, and potentially cost-effective intervention for healthcare workers addressing a number of diseases and creating a diverse array of interventions with varying degrees of efficacy and reproducibility [16]–[21]. Our mobile phone application, *Mobilize,* electronically standardizes the recording and tracking of adverse events experienced by MDR-TB patients and utilizes simple, low-cost, functional phones. It also provides clinical decision aids for triaging emergent cases requiring attention and facilitates real-time HCW-physician communication through automatic data transfers from phones in the field to the physicians’ desk, reducing the cost barrier of phone calls and communication delays.

*Mobilize* was the first known introduction of mHealth in this resource-limited area. We performed a pragmatic trial, evaluating the feasibility and acceptability of the intervention in everyday clinical practice and allowing for adaptation to local needs [22]–[25], and present a mixed-methods assessment of piloting *Mobilize* at one decentralized treatment center in rural KZN, the Greytown Specialized Drug-Resistant TB Treatment Hospital (GTN).

## Methods

### Ethical Review

Institutional review boards at the University of Michigan Medical School, Yale University School of Medicine, and the University of KwaZulu-Natal Biomedical Research Ethics Committee approved this study.

### Study Site

KZN has a population of over 10 million, consisting largely of poor, uneducated individuals, whose 2011 life-expectancy at birth was 57.1 years [26]. Our work takes place in the Umzinyathi District of KZN, which hosts GTN, the first of four decentralized treatment centers in KZN. Each center treats a similar number of MDR-TB patients annually, have comparable demographics–treating the poorest individuals in the province, structure their community-based treatment models similarly, and have outcomes similar to the traditional, centralized, tertiary-care hospital [5], [27]. Given the pilot nature of our study, we focused our intervention to an Umzinyathi’s sub-district already using GTN for MDR-TB care coordination. Mobile HCWs are trained health professionals employed by the KZN Department of Health who are a critical part of decentralized care. They are trained to administer an injectable TB therapy at home, triage symptoms in the field, transfer patients urgently to an inpatient facility, and trace (i.e. track down) MDR-TB patients who are either newly diagnosed or are defaulting (e.g. missing appointments or not adhering to medications).

### Mobilize Study Overview

**Figure 1:**
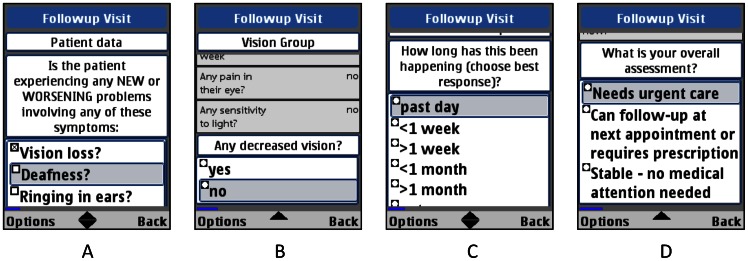
Screen shot images of *Mobilize* on the mobile phones.

**Figure 2:**
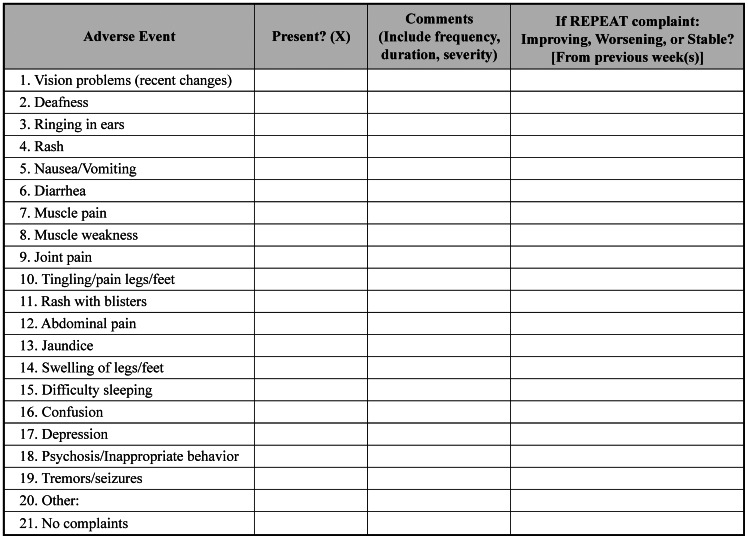
Traditional paper adverse events forms.

The mobile phone application, *CommCare* [21], [28], developed by Dimagi, Inc., was iteratively modified into *Mobilize* (**Figure 1**) for use during the study period of April to October 2011. The standard KZN DOH paper forms (**Figure 2**) served as the basic structure for the initial phone application. After feedback sessions with mobile HCWs and community clinicians, *Mobilize* was further modified for improved functionality and incorporated newly suggested components: (1) decision aids for triaging symptom complaints; (2) KZN DOH adherence questions; and (3) a tool for tracing newly diagnosed TB patients, both standard and drug-resistant, or finding defaulters from TB treatment.

Once the application was finalized, two investigators (K.H.C. and M.L.) conducted an on-site, two-day training session with all five of the active mobile HCWs employed at GTN, including one-on-one in-field training with investigators prior to the beginning of the study period. One head nurse and one data manager was trained to organize the day-to-day operations for *Mobilize* and provided local oversight. Additionally, technical and logistical support was continuously available and provided on an as needed basis. These were further addressed during scheduled conference calls every two weeks and regular email communication.

Throughout the course of the seven-month study period airtime, text, data for Internet use, car-based cellular chargers, and feature phones (Nokia® 2700 and 2730 models) were supplied free of charge to the five HCWs. In South Africa, airtime, text, and data plans are typically purchased separately and are pre-paid until the monetary amount is completely utilized or the purchase expires at the end of the month. Therefore, we coordinated pre-paid purchases of airtime, text messaging, and data that totaled $11 USD and allowed each phone to receive two additional recharges, or top-ups, to meet estimated demands for the phone’s various functions, totaling a maximum of $33 USD per phone per month. A similar phone plan was allocated for the phones held by the onsite managers. Data entered on the phone was automatically uploaded to a secure online database for unlimited access by the health professionals and could be printed at any time point for the patient’s paper record.

In accordance with South Africa’s national drug-resistant TB treatment guidelines, mobile HCWs were asked to enter adverse events weekly for each patient during the intensive phase – when patients receive a daily injectable aminoglycoside for approximately 6 months in a 24-month treatment course [14]. We limited our intervention to patients in the intensive phase because adverse events are more typically experienced during this period compared with the remainder of the treatment course [29]. Traditional paper forms continued to be available, but mobile HCWs were encouraged to primarily use *Mobilize* and carry paper forms for recording in the case of a technical malfunction. The physician and nursing staff based on-site at GTN regularly reviewed and addressed voice calls, text messages, and forms from the online database. Patients continued to attend their monthly follow-up appointments at the GTN clinic. Each mobile HCW received a mobile phone and the GTN-based staff shared one.

### Sampling & Recruitment

We qualitatively interviewed all five mobile HCWs. One investigator (K.H.C.) explained the project and obtained written consent from participants. All verbal and written information was delivered in English, a language requirement for all DOH clinical employees.

### Analytical Methods

A mixed-methods analysis was chosen to evaluate this pragmatic trial for the purpose of informing policymakers at the KZN DOH [20], [22], [25], [30], [31].

### Quantitative

#### Study outcomes

The primary feasibility outcome was the proportion of weekly adverse events forms submitted versus expected by mobile HCWs. The expected number of forms was based on the weekly number of GTN patients in the intensive phase of MDR-TB therapy who were already being actively tracked and managed through the pre-existing decentralized MDR-TB treatment program. Patients entered the expected form pool if they were in the intensive phase of therapy at the start of the study period or started the intensive phase during the study period. Patient data was censored if they were not at home during the intensive phase (e.g. admitted as an inpatient), died during the study period, transitioned to the continuation phase, or moved into another sub-district. A baseline expected form submission percentage was calculated for patients undergoing the intensive phase of therapy between April 2010 and April 2011. Individual HCW submission patterns were not analyzed as mobile HCWs rotated which patients they saw on a daily basis. Secondary results included perceived comfort levels with using mobile phone technology as a measure of acceptability, the quality of adverse events monitoring, and the proportion of reportable adverse events being captured. These were assessed in a questionnaire administered before implementation of *Mobilize* with all the HCWs in the study, and then repeated at four months and after study completion. The questionnaire assessed comfort using mobile phones for adverse events monitoring (Likert scale), the quality of adverse events reporting (Likert scale) and the percentage of adverse events being captured (visual analogue scale, VAS) through *Mobilize*. Statistical comparisons were not made given the small sample size of our study.

#### Technical Outcomes

Phone usage patterns were tracked based on how often money was being replenished into each phone. However, delineating whether the phone was used for personal matters or patient care was not technically feasible and recording phone numbers dialed or texted could be inaccurate as mobile HCWs might dial or text a variety of community members to contact or track a patient. We recorded any technical problems experienced by the mobile phones or the *Mobilize* application. All descriptive data was analyzed using STATA v11 (StataCorp, College Station, TX).

### Qualitative

#### Data Collection

Qualitative data was collected through two in-depth focus group interviews, completed four months after the study start date and within two months post-study. Interviews consisted of open-ended questions designed to address the following topics: (1) perceptions on using mobile-phone technology for monitoring adverse events, adherence, and tracing, and (2) impacts of the mobile-phone technology on the personal work environment. All interviews were conducted by K.H.C. or M.L. in private locations (where conversations could not be overheard), audio-recorded with permission, and averaged an hour in length per interview.

### Data Preparation & Analysis

South African research staff members transcribed the interviews from audio recordings in pairs and then compared their results for quality. K.H.C. and M.L. addressed discrepancies. Qualitative analysis was directed towards developing an explanation of the perceived quality of adverse events monitoring and the evolving experiences of using mHealth technology. K.H.C. and M.L. reviewed all transcriptions for analysis, creating a codebook to categorize responses into major themes and perform line-by-line coding of transcripts.

## Results

### Participant Characteristics

One of five mobile HCWs was female; the median age was 32 (range 27–46) years for all HCWs. The HCWs were employed by the DOH for a median of 6 years (range 0.5–18) and had a median 3 years (range 0.5–5) of experience with MDR-TB patients. Two mobile HCWs were nurses; three completed secondary schooling without additional training. Pre-study, their median comfort level with mobile devices was 8 (range 3–10, [0 = “Not at all” to 10 = “The most comfortable I will ever feel”]).

### Quantitative Results

#### Study outcomes

Within the seven-month study period, four total patients were undergoing the intensive phase of treatment. Mobile HCWs electronically submitted nine of 33 (27%) total expected weekly adverse events forms (**Table 1**). Thirty-three expected forms is calculated based on two patients who were in the intensive phase for four weeks during the study period, one for seven weeks, and one for 18 weeks. In comparison, 14 of 299 (5%) expected paper forms were submitted for 16 patients, who had variable amounts of time in the intensive phase, during the year prior to our intervention. No paper forms were submitted during our study period and, to our knowledge, the reporting of an adverse event was not hampered by the absence of a traditional paper form being available. Likert scale surveys found that most mobile HCWs consistently felt comfortable using mobile phones for adverse events monitoring and agreed that the quality of the adverse events reported was good to excellent; however, none of the follow-up VAS surveys indicated that all adverse events were being captured during the study period.

**Table 1:** Quantitative data.

	Baseline	4 month	7 month
Proportion of forms submitted, actual (%)	14/299* (5%)	–	9/33 (27%)
Comfort with mobile phones (range)	8 (3–10)	7 (5–10)	8 (7–9)
Quality of AEs captured (range)	3 (3–3)	3 (2–5)	4 (2–5)
Percent of AEs captured, subjective (range)	100 (30–100)	60 (50–80)	55 (30–70)

Comfort was based on a 10-point Likert scale [0 = “Not at all” to 10 = “The most comfortable I will ever feel”]). Quality was based on a 5-point Likert scale [0 = “Poor” to 5 = “Excellent”]. The subjective percent of AEs captured was based on a VAS scale ranging from 0% (none) to 100% (all).

* Based on data collected from April 2010 to April 2011, the year prior to the study period.

#### Technical outcomes

A proxy for usage patterns was assessed using the proportion of available airtime, SMS, and data bundles that were refilled during the study period –55%, 33% and 18%, respectively. Mobile HCWs were unable to log into the *Mobilize* application twice because the login screen froze. This issue was fixed twice, within one week, by deleting and re-uploading a new version of the application. To our knowledge, there were no reported problems with uploading the data because of poor cellular coverage or slow upload speeds. One mobile phone malfunctioned, which was replaced within one week. No phones were stolen or missing at the end of the study.

### Qualitative Results

#### Overview

Results from focus group interviews conflicted with the low return rate of adverse events forms. Mobile HCWs’ comments fell under four separate themes: (1) *Mobilize* improved communication; (2) *Mobilize* improved their workflow; (3) *Mobilize* could be expanded to help with other work related tasks; and (4) challenges occurred with using the phone and phone application.

#### Theme 1: Mobilize improved communication

Mobile HCWs noted how communication was increased with patients and GTN-based staff, keeping them all in touch while they were driving on the roads. Though they have always had personal phones for use, mobile HCWs were reluctant to use them for patient care because of the cost.

*“It is a very useful to have [the Mobilize phones] because at times…you need to get hold of a [GTN staff members] now. Eish! With the pay that we are getting we can’t afford to phone the landline during the day [with our own phones] and often we wait for off peak times to phone… with [the Mobilize phones] you just phone anytime.”*

By being able to communicate, they were able to provide more timely patient care. Compared with paper forms one HCW stated, “We are reporting immediately as soon as we’re done, that means that the continuity of care with the patient is not being disrupted in any way.” Voice calls and text messaging functions were highly regarded as they enabled collaboration when trying to find a patient and reduced travel time. “We don’t have to [go to GTN] all the time to report everything, it was done [over the phone] and it made it easier for [GTN] to contact us and us to contact them,” stated one mobile HCW.

Moreover, after spending most of their days on the road, the phones help the mobile HCWs feel more incorporated into the team.

*“With [this phone] we are able to work as a team, this is a team phone…it has made communication between the team quite good and also for the patients because if you don’t communicate as a team…they are getting less care than they should.”*

This team perspective was enhanced because mobile HCWs felt the information transmitted with the *Mobilize* phone application was more clinically useful for the patients compared with the traditional paper forms. “Who was going to read the [paper] form? We know [GTN staff] have received [the phone submission] when you meet the [patient] after a clinic appointment and they tell you they had [a medical issue] and then you see that [the phone] has been effective,” stated one mobile HCW.

Many also felt that using mobile phones was a safer way to store and transmit patient information compared with traditional paper forms. One individual remarked:

*“Rather than carrying a whole lot of papers, you just carried a phone, it had all that we needed instead of those papers…we lose them and we lost them under the car seats or on the floor and you find them everywhere…[the phone] is always in your pocket.”*

#### Theme 2: Mobilize improved workflow

With regards to the three main features of the *Mobilize* phone application – adverse events reporting, monitoring, and tracing, the mobile HCWs had a positive perception of the application’s usefulness. Overall, they felt that increasing the frequency of adverse events monitoring was beneficial - “You are able to report if the patient complains of anything and…we are doing it weekly so it is better that way.”

Mobile HCWs believed they were previously able to avoid adverse events monitoring because of limited oversight with the traditional paper forms. With the phones, one individual believed they felt like they were being monitored and more inclined to conduct adverse events monitoring more frequently. One person stated “were unable to neglect this form when we are using [the phone].”

The tracing component of the application built into the *Mobilize* application was often mentioned as a positive feature because it became “logistically simpler” and turn around times were improved from three to four days down to one day between tracing request and finding a patient in the field.

#### Theme 3: Expanding the scope of mobilize

The mobile HCWs had a number of suggestions about how *Mobilize* could benefit other health programs in the GTN area. As an example, they often questioned why the scope was limited to only MDR-TB patients. Their personal tasks were not limited to MDR-TB patients; therefore, they expanded the use of the voice and text messaging functions to caring for other patient populations, like drug-sensitive TB and HIV patients. Further to this point, one mobile HCW stated, “I think the phones would be helpful if not only looks at TB. It should also look at other diseases or problems…like immunizations and ante-natal care.” Another mobile HCW believed utilizing the phone application for all TB patients might prevent the development of resistance:

*“I feel that one of the predisposing causes of the [MDR-TB] is improper management, so giving us a platform to actually communicate so that we are trying to prevent the patient from going into the MDR/XDR stage…So, we use the phone [for normal TB patients]…The purpose is to prevent the patient from progressing into the stage that is quite difficult to treat…[the phone] is not treatment. It is communication. It will make quite a big difference.”*

#### Theme 4: Challenges

The phone experienced technical issues that were related to the program periodically freezing which occurred at least once every two weeks. At times when users were trying to access the *Mobilize* program, they were unable to upload data needed for the day, resulting in patient care delays. As one worker stated:

*“The application on the phone is time consuming at times because you are waiting to log, log, log…We are talking about three to five minutes waiting for [the program to start], instead of chatting with someone.”*

As a result, one user suggested, “I would like [DOH] to hire a technician or a required person who would be dealing with the phones 24 hours, 24/7.” Otherwise, HCWs felt the basic functions of the phone, like voice calls and texts, worked without problems.

Moreover, mobile HCWs felt limited when discussing the money allocated for airtime, as voice calls during daytime working hours tend to be the most expensive. As a result, some believed they were “doing a rush job through that call because we got to save it for other patients too.”

#### Conflicting results

Despite the overall positive reception towards implementing mHealth in their daily workflow and the positive response to having the phones and *Mobilize* available, the program was used very little for the primary aim of the project, adverse events monitoring. When asked directly, mobile HCWs apologized at each focus group for not having had used the *Mobilize* application more often during the study period, often stating they had forgotten.

Moreover, in attempts to explain why forms – paper or electronic, were not being completed as rigorously, mobile HCWs generally felt that clinical staff should impart more patient responsibility when discussing adherence. As one HCW states:

*“One of the reasons for adherence counseling is for them to be able to take informed decisions about their lives and not for us to always be doing things for them because at the end of the day, TB treatment will be over, we will be gone, but they still need to be good. We don’t need to baby sit them all the time, they need to take responsibility.”*

## Discussion

We developed a pilot mHealth intervention, *Mobilize,* to address inadequate reporting of adverse events and improve follow up of MDR-TB patients being treated in a community-based program in rural KZN. A low cost (**Table 2** **)** phone application was created using an iterative design process, successfully deployed, and qualitative feedback from our end-users (i.e. mobile HCWs) was positive. Mobile HCWs expressed enthusiasm for the product because it facilitated a sense of team between themselves and clinic-based staff, while also allowing them to communicate more readily and frequently with patients and not incur any personal financial costs. Moreover, the ability to trace non-adherent or patients lost-to-follow-up was well received because they no longer had to drive back to GTN for notifications and could receive the requests remotely. Though we cannot account for temporal trends, the submission rate of adverse events generally improved from paper forms (5%) to or mHealth application (27%), but both methods resulted in poor submission rates. If mHealth is to play a more critical role in care delivery, understanding the needs and interests of healthcare workers using mHealth tools will be important for uptake so public health departments can evaluate whether mHealth technology helps achieve program objectives. Additionally, assessments of mHealth interventions require objective outcomes, not simply indications of acceptability.

**Table 2:** Cost data.

Items		Cost (USD)
Training sessions		163.48
Project supplies (computer, copying, printing, etc.)		1,565.15
Mobile phones devices*		1,073.66
Phone usage by HCWs^**^		632.26
	**Total**	**$3,434.55**

Does not include the costs of salaries of personnel, travel, or living expenses. All programming was done voluntarily by one of the investigators (SL). Dimagi provided the CommCare platform for free.

* Nine total phones were purchased, a number were backup phones for any potential technical malfunctions. **Phone usage costs were comprised of airtime ($425.72), SMS ($128.45), and data costs ($78.09).

One explanation offered by mobile HCWs for forgetting to check adverse events was an underlying sense that patients should have more responsibility in their care. Future interventions for improving adverse event monitoring should focus on understanding the root cause of why HCWs may not be using mHealth expected - whether the limitation is a structural barrier, lack of personal motivation, or multifactorial. Technologic innovations and applications are prone to the limitations encountered when trying to change worker behaviors or beliefs. Despite being a part of their job description, our HCWs believed patients, not mobile HCWs, should be held responsible for reporting their symptoms. Though not within the scope of this project, a deeper understanding of the relationship between HCWs and patients within the cultural context of our setting might be beneficial before health programs develop interventions designed to improve clinical care. Furthermore, the response of “I have forgotten” towards questions of accountability should be interpreted with caution. It is plausible there are other reasons for not reporting which we did not explore, and “forgetting” maybe a proxy for alternative explanations. For instance, mobile HCWs might have been reluctant to tell investigators due to a social desirability bias to garner our approval.

Exploring features of the phone or technology perceived to be of greatest utility might have helped set more realistic expectations of uptake. *Mobilize* was implemented with the intent of providing a phone application focused on improving the ease and efficiency of adverse events monitoring, but HCWs found more benefit in being able to complete basic text and voice calls with co-workers and patients. Holden et al explores the complexity of uptake within the Technology Acceptance Model for information technology in healthcare, the perceived usefulness of technology ultimately depends on what the HCW deems important [32]. The ease of use does not correlate with the perception of a technology’s usefulness, though having HCWs confidently able to use technology is a pre-disposing step towards usefulness. We focused on developing and customizing the phone application towards improving adverse events monitoring and helping HCWs gain confidence in using mobile technology, but improving adverse events was not what HCWs believed to be the most useful component of the mobile phones provided. Based on usage patterns, HCWs used the phone mostly for text and voice communication given those features were monetarily replenished regularly throughout the intervention period. Additionally, in the iterative design process, clinical providers might have diluted the perceptions of mobile HCWs because they have a different vantage point regarding the use and function of the mobile phones. Stronger consideration of end-user perspectives may improve the uptake of future mHealth applications.

Moreover, mobile HCWs believed mobile phone interventions should expand the scope beyond disease specific programs. Their daily tasks are not limited to only MDR-TB patients. Mobile HCWs track and visit patients throughout the district who have missed primary care, have HIV, or require additional home visits. At the time of our study, the KZN Department of Health separated their public health programs based on disease. For example, the administrative oversight of TB was separated from the HIV programs; yet, our mobile HCWs were asked to be a part of both. Therefore, when considering their everyday work environment – driving across an expansive terrain, communicating with multiple administrators and other clinical staff, mobile HCWs may have seen our efforts to improve adverse events as another burdensome task by a disease specific administrator [9], [33]. Similarly, the consideration of everyday, real-world work constraints should be extended to clinicians and support staff involved in implementing our mHealth intervention, as their tasks often expanded beyond MDR-TB to primary care program needs. Future mHealth interventions should explore the total work environment needs for the total healthcare workforce and consider interventions that meet the complexity of their work demands. Considering this framework it is not unexpected that text and voice calls were the most useful mobile phone features from the outset.

In line with previous studies, the conflict between high interest and low usage of mHealth has been observed in other resource-limited settings. Haberer et al [18] found high participation interests for using mHealth to measure antiretroviral therapy adherence in rural Uganda using interactive voice response and short messaging service, but the completion of adherence queries was less than 33%. The major perceived limitation in achieving better completion rates was due to a technical misunderstanding of how to use mHealth technology, although motivation may have also played a role. In the *Mobilize* study, we provided in-depth training for a well-educated user population prior to implementation and technical support throughout, but we had similarly low user uptake.

Though we have learned a number of important lessons, our results should be interpreted cautiously because of the small number of mobile HCWs and the technical limitations of our pilot study. We do not know, for example, how uptake might have changed over time or the impact of having regular oversight from local, more senior clinical staff. Conclusions from this study site may not be generalizable to other areas because the demographics of our mobile HCWs; for example a more predominantly female HCW cohort might have had higher uptake [34]. We were unable to track the technical barriers our mobile HCWs might have experienced like electronic submission errors or screen freezes, because we did not have the technical capacity to track such errors electronically nor did we incorporate a manual recording system in our study design. Detailed usage patterns, like whether the phone was used more for personal matters versus work-related tasks, could not be recorded because we lacked the technical capacity to track them also. Closer, real-time monitoring and evaluation of staff performance might improve uptake of mHealth interventions and work performance.

In conclusion, mHealth interventions are a complex interplay between the motivations of the end-user and the functional applicability of the technology in everyday work environments. We were able to successfully train and deploy a customized mHealth program in a rural, resource-poor, sub-Saharan Africa setting with high acceptability and buy-in from clinical coordinators and health professionals but fell short of our goal to feasibly improve the quantity of adverse events monitoring. Current efforts are underway in rural KZN to strengthen the total public health infrastructure. If mHealth is to assist, research should further explore the motivations of HCWs within the context of their workflow limitations and improved technology for closer, real-time performance monitoring to create a scalable intervention that is more likely to improve our awareness of adverse events that have occurred and then ultimately turn our focus towards properly managing these events.

## References

[1] World Health Organization (2012) Global tuberculosis control 2012. Geneva: World Health Organization.

[2] Gandhi NR, Andrews JR, Brust JC, Montreuil R, Weissman D, et al (2012) Risk factors for mortality among MDR- and XDR-TB patients in a high HIV prevalence setting. Int J Tuberc Lung Dis 16: 90–97. 22236852 10.5588/ijtld.11.0153 PMC3302205

[3] Gandhi NR, Shah NS, Andrews JR, Vella V, Moll AP, et al (2010) HIV coinfection in multidrug- and extensively drug-resistant tuberculosis results in high early mortality. Am J Respir Crit Care Med 181: 80–86. 19833824 10.1164/rccm.200907-0989OC

[4] Brust JC, Shah NS, Scott M, Chaiyachati K, Lygizos M, et al (2012) Integrated, home-based treatment for MDR-TB and HIV in rural South Africa: an alternate model of care. Int J Tuberc Lung Dis 16: 998–1004. 22668560 10.5588/ijtld.11.0713 PMC3390442

[5] Loveday M, Wallengren K, Voce A, Margot B, Reddy T, et al (2012) Comparing early treatment outcomes of MDR-TB in decentralised and centralised settings in KwaZulu-Natal, South Africa. Int J Tuberc Lung Dis 16: 209–215. 22236922 10.5588/ijtld.11.0401 PMC3281510

[6] Padayatchi N, Friedland G (2008) Decentralised management of drug-resistant tuberculosis (MDR- and XDR-TB) in South Africa: an alternative model of care. Int J Tuberc Lung Dis 12: 978–980. 18647461

[7] Loveday M, Thomson L, Chopra M, Ndlela Z (2008) A health systems assessment of the KwaZulu-Natal tuberculosis programme in the context of increasing drug resistance. Int J Tuberc Lung Dis 12: 1042–1047. 18713502

[8] Brust JC, Lygizos M, Chaiyachati K, Scott M, van der Merwe TL, et al (2011) Culture conversion among HIV co-infected multidrug-resistant tuberculosis patients in Tugela Ferry, South Africa. PLoS One 6: e15841. 21253585 10.1371/journal.pone.0015841 PMC3017058

[9] Loveday M, Zweigenthal V (2011) TB and HIV integration: obstacles and possible solutions to implementation in South Africa. Trop Med Int Health. 10.1111/j.1365-3156.2010.02721.x 21255204

[10] Shin SS, Pasechnikov AD, Gelmanova IY, Peremitin GG, Strelis AK, et al (2007) Adverse reactions among patients being treated for MDR-TB in Tomsk, Russia. Int J Tuberc Lung Dis 11: 1314–1320. 18034952

[11] Ammassari A, Antinori A, Aloisi MS, Trotta MP, Murri R, et al (2004) Depressive symptoms, neurocognitive impairment, and adherence to highly active antiretroviral therapy among HIV-infected persons. Psychosomatics 45: 394–402. 15345784 10.1176/appi.psy.45.5.394

[12] Ammassari A, Murri R, Pezzotti P, Trotta MP, Ravasio L, et al (2001) Self-reported symptoms and medication side effects influence adherence to highly active antiretroviral therapy in persons with HIV infection. J Acquir Immune Defic Syndr 28: 445–449. 11744832 10.1097/00042560-200112150-00006

[13] Starace F, Ammassari A, Trotta MP, Murri R, De Longis P, et al (2002) Depression is a risk factor for suboptimal adherence to highly active antiretroviral therapy. J Acquir Immune Defic Syndr 31 Suppl 3S136–139. 12562037 10.1097/00126334-200212153-00010

[14] South African Department of Health (2009) Guidelines for the Management of Drug-resistant TB 2009. Pretoria: South African Department of Health. 109.

[15] Al-Dakkak I, Patel S, McCann E, Gadkari A, Prajapati G, et al.. (2012) The impact of specific HIV treatment-related adverse events on adherence to antiretroviral therapy: A systematic review and meta-analysis. AIDS Care. 10.1080/09540121.2012.712667 PMC3613968 22908886

[16] Zurovac D, Sudoi RK, Akhwale WS, Ndiritu M, Hamer DH, et al (2011) The effect of mobile phone text-message reminders on Kenyan health workers' adherence to malaria treatment guidelines: a cluster randomised trial. Lancet 378: 795–803. 21820166 10.1016/S0140-6736(11)60783-6 PMC3163847

[17] Hoffman JA, Cunningham JR, Suleh AJ, Sundsmo A, Dekker D, et al (2010) Mobile direct observation treatment for tuberculosis patients: a technical feasibility pilot using mobile phones in Nairobi, Kenya. Am J Prev Med 39: 78–80. 20537846 10.1016/j.amepre.2010.02.018

[18] Haberer JE, Kiwanuka J, Nansera D, Wilson IB, Bangsberg DR (2010) Challenges in using mobile phones for collection of antiretroviral therapy adherence data in a resource-limited setting. AIDS Behav 14: 1294–1301. 20532605 10.1007/s10461-010-9720-1 PMC2975780

[19] Lester RT, Ritvo P, Mills EJ, Kariri A, Karanja S, et al (2010) Effects of a mobile phone short message service on antiretroviral treatment adherence in Kenya (WelTel Kenya1): a randomised trial. Lancet 376: 1838–1845. 21071074 10.1016/S0140-6736(10)61997-6

[20] Chang LW, Kagaayi J, Arem H, Nakigozi G, Ssempijja V, et al (2011) Impact of a mHealth intervention for peer health workers on AIDS care in rural Uganda: a mixed methods evaluation of a cluster-randomized trial. AIDS Behav 15: 1776–1784. 21739286 10.1007/s10461-011-9995-x PMC3265752

[21] Derenzi B, Borriello G, Jackson J, Kumar VS, Parikh TS, et al (2011) Mobile phone tools for field-based health care workers in low-income countries. Mt Sinai J Med 78: 406–418. 21598267 10.1002/msj.20256

[22] Jansen YJ, Foets MM, de Bont AA (2010) The contribution of qualitative research to the development of tailor-made community-based interventions in primary care: a review. Eur J Public Health 20: 220–226. 19561172 10.1093/eurpub/ckp085

[23] Treweek S, Zwarenstein M (2009) Making trials matter: pragmatic and explanatory trials and the problem of applicability. Trials 10: 37. 19493350 10.1186/1745-6215-10-37 PMC2700087

[24] Zwarenstein M, Treweek S (2009) What kind of randomized trials do we need? J Clin Epidemiol 62: 461–463. 19348970 10.1016/j.jclinepi.2009.01.011

[25] Tunis SR, Stryer DB, Clancy CM (2003) Practical clinical trials: increasing the value of clinical research for decision making in clinical and health policy. JAMA 290: 1624–1632. 14506122 10.1001/jama.290.12.1624

[26] (2011) Mid-Year Population Estimates, 2011. Statistics South Africa.

[27] Loveday M, Wallengren K, Voce A, Margot B, Master I, et al. Treatment outcomes in decentralised model of care for MDR-TB patients in KwaZulu-Natal, South Africa.; 2012 June 12–15, 2012; Durban, South Africa.

[28] Dimagi. Available: www.dimagi.com. Accessed 2013 April 22.

[29] Isaakidis P, Varghese B, Mansoor H, Cox HS, Ladomirska J, et al (2012) Adverse events among HIV/MDR-TB co-infected patients receiving antiretroviral and second line anti-TB treatment in Mumbai, India. PLoS One 7: e40781. 22792406 10.1371/journal.pone.0040781 PMC3394731

[30] Godwin M, Ruhland L, Casson I, MacDonald S, Delva D, et al (2003) Pragmatic controlled clinical trials in primary care: the struggle between external and internal validity. BMC Med Res Methodol 3: 28. 14690550 10.1186/1471-2288-3-28 PMC317298

[31] Creswell JW (2009) Research Design: Qualitative, Quantitative, and Mixed Methods Approaches. Los Angeles: Sage Publications, Inc. 260 p.

[32] Holden RJ, Karsh BT (2010) The technology acceptance model: its past and its future in health care. J Biomed Inform 43: 159–172. 19615467 10.1016/j.jbi.2009.07.002 PMC2814963

[33] Doherty T, Chopra M, Nsibande D, Mngoma D (2009) Improving the coverage of the PMTCT programme through a participatory quality improvement intervention in South Africa. BMC Public Health 9: 406. 19891775 10.1186/1471-2458-9-406 PMC2777166

[34] Grameen Foundation (2011) Mobile technology for community health in Ghana: What it is and what Grameen Foundation has learned so far. Available: http://www.grameenfoundation.org/sites/default/files/MOTECH-Early-Lessons-Learned-March-2011-FINAL.pdf. 85 p. Accessed 2013 April 13.

